# Supplementary material for: Is increased mortality by multiple exposures to COVID-19 an overseen factor when aiming for herd immunity?
Source: PLoS One. 2021 Jul 16;16(7):e0253758. doi: 10.1371/journal.pone.0253758 (PMC8284653; doi:10.1371/journal.pone.0253758)
Supplement: S1 Fig — The same as Fig 2 but without seasonal fluctuations. (PDF) [file pone.0253758.s001.pdf]

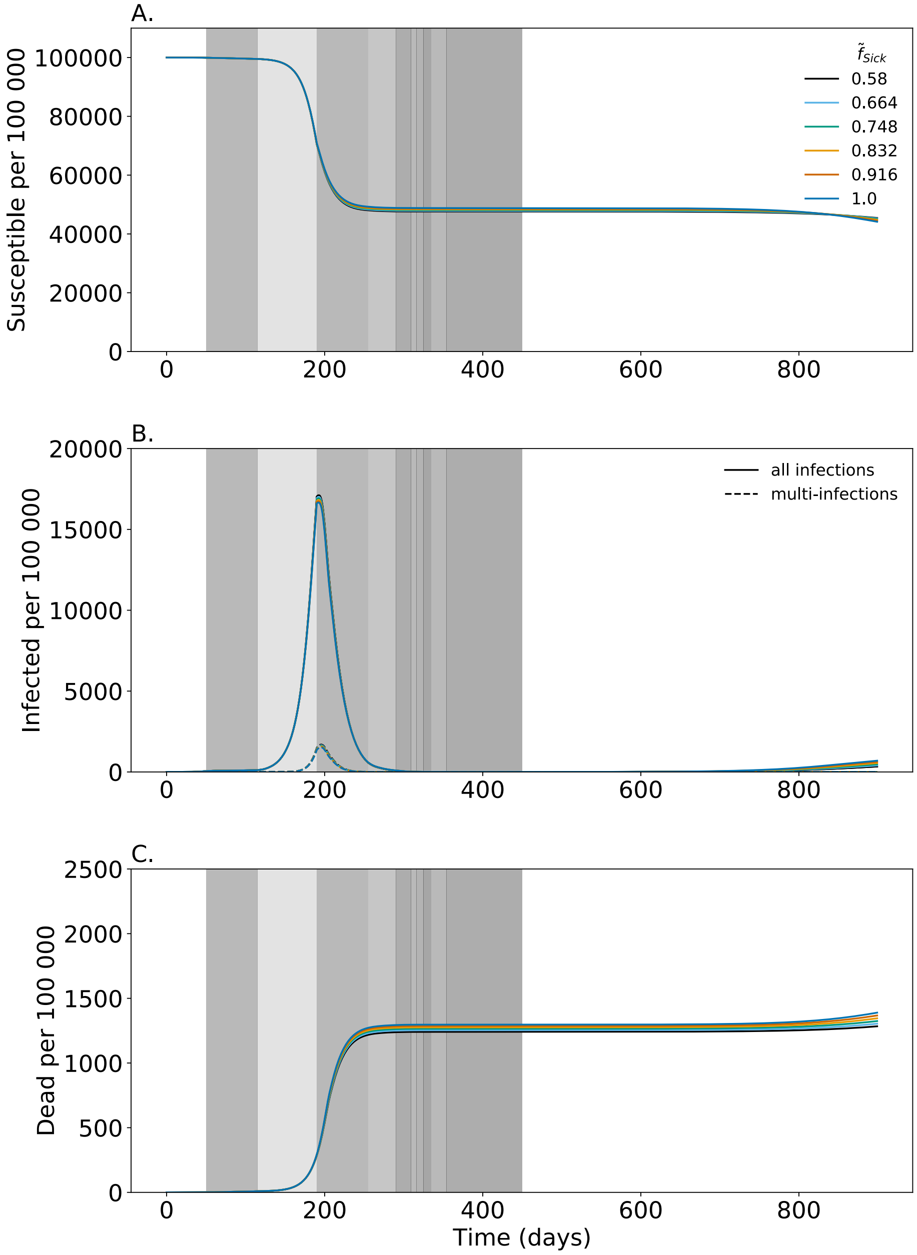

S1 Fig. Morbidity caused by multi-infections without seasonal fluctuations. The same as Fig 2 but without seasonal fluctuations.
