# Supplementary material for: Is increased mortality by multiple exposures to COVID-19 an overseen factor when aiming for herd immunity?
Source: PLoS One. 2021 Jul 16;16(7):e0253758. doi: 10.1371/journal.pone.0253758 (PMC8284653; doi:10.1371/journal.pone.0253758)
Supplement: S2 Fig — Shown are the total numbers of (A) deaths; (B) case fatality (i.e., deaths per infected individuals); (C) total percentage (of the population) being infected or multi-infected; and (D) the maximal number of individuals infected or multi-infected at certain census points. For each value of general distancing pDist after day t = 450 values are shown under the assumptions that: (a) multi-infections cause higher morbidity and mortality, and are more likely to be isolated than single-infections (corresponding to Fig 7A–7C); (b) morbidity, mortality, and isolation are the same as for single-infections (corresponding to Fig 7D–7F). The horizontal lines in scenario (a) in (C) and (D) indicate the number of multi infections characterized by increased morbidity and mortality given by (33) in S1 Appendix. Parameters are given in S4 and S5 Tables. (PDF) [file pone.0253758.s002.pdf]

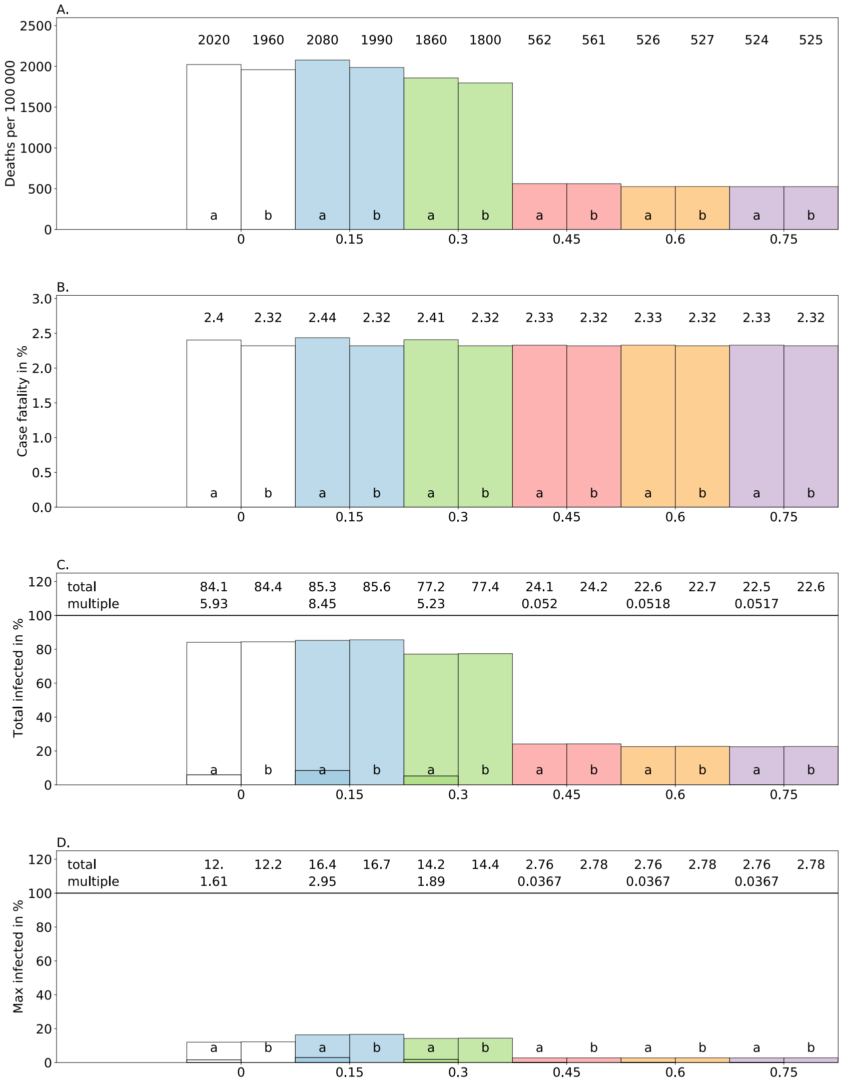

S2 Fig. Characteristics of multi-infections. Shown are the total numbers of (A) deaths; (B) case fatality (i.e., deaths per infected individuals); (C) total percentage (of the population) being infected or multi-infected; and (D) the maximal number of individuals infected or multi-infected at certain census points. For each value of general distancing p_{Dist} after day $t = 450$ values are shown under the assumptions that: (a) multi-infections cause higher morbidity and mortality, and are more likely to be isolated than single-infections (corresponding to Fig 7A-C); (b) morbidity, mortality, and isolation are the same as for single-infections (corresponding to Fig 7D-F). The horizontal lines in scenario (a) in (C) and (D) indicate the number of multi infections characterized by increased morbidity and mortality given by (33) in S1 Appendix. Parameters are given in S4 Table –S5 Table.
