# Supplementary material for: Is increased mortality by multiple exposures to COVID-19 an overseen factor when aiming for herd immunity?
Source: PLoS One. 2021 Jul 16;16(7):e0253758. doi: 10.1371/journal.pone.0253758 (PMC8284653; doi:10.1371/journal.pone.0253758)
Supplement: S1 Table — (PDF) [file pone.0253758.s004.pdf]

S1 Table. Population size and model compartments.

Name	Description	Value
N	Population size	331 000 000
$S(0)$	No. susceptible	330 999 925
$E_k(0)$	No. single-infected in kth latent states ($1 \leq k \leq n_E$)	0
$E^*_k(0)$	No. transient multi-infections in latent states ($1 \leq k \leq n_E$)	0
$\tilde{E}_k(0)$	No. multi-infected in kth latent states ($1 \leq k \leq n_E$)	0
$P_k(0)$	No. single-infected in kth prodromal states ($1 \leq k \leq n_P$)	0
$P^*_k(0)$	No. transient multi-infections in prodromal states ($1 \leq k \leq n_P$)	0
$\tilde{P}_k(0)$	No. multi-infected in prodromal states ($1 \leq k \leq n_P$)	0
$I_1(0)$	No. single-infected in first fully contagious Erlang state	75
$I_k(0)$	No. single-infected in kth fully contagious states ($2 \leq k \leq n_I$)	0
$I^*_k(0)$	No. transient multi-infections in kth fully contagious states ($1 \leq k \leq n_I$)	0
$\tilde{I}_k(0)$	No. multi-infected in full contagious states ($1 \leq k \leq n_I$)	0
$L_k(0)$	No. single-infected in kth late-infectious states ($1 \leq k \leq n_L$)	0
$L^*_k(0)$	No. transient multi-infections in kth late-infectious states ($1 \leq k \leq n_L$)	0
$\tilde{L}_k(0)$	No. multi-infected in kth late-infectious states ($1 \leq k \leq n_L$)	0
$R(0)$	No. recovered	0
$D(0)$	No. dead	0

Population size, model compartments and their respective parameter choices.
