# Supplementary material for: Is increased mortality by multiple exposures to COVID-19 an overseen factor when aiming for herd immunity?
Source: PLoS One. 2021 Jul 16;16(7):e0253758. doi: 10.1371/journal.pone.0253758 (PMC8284653; doi:10.1371/journal.pone.0253758)
Supplement: S2 Table — (PDF) [file pone.0253758.s005.pdf]

S2 Table. Summary of model parameters describing disease progression and choices for the simulations.

Name	Description	Value
n_{E}	No. of latency Erlang states	16
n_{P}	No. of prodromal Erlang states	16
n_{I}	No. of fully contagious Erlang states	16
n_{L}	No. of late-infectious Erlang states	16
D_E	Average duration of latency period	3.7 days
D_P	Average duration of prodromal period	1 day
D_I	Average duration of fully contagious period	5 days
D_L	Average duration of late infectious period	5 days
ε	Transition rate of latent states	n_{E}/D_E
φ	Transition rate of prodromal states	n_{P}/D_P
γ	Transition rate of early infectious states	n_{I}/D_I
δ	Transition rate of late-infectious states	n_{L}/D_L
α	Transition rate from transient multi-infections to multi-infected states	3.2/day
f_{Sick}	Fraction of symptomatic (sick) infections	58%
$\tilde{f}_{\mathrm{Sick}}$	Fraction of sympt. multi-infections in fully contagious & late inf. periods	64.4%
f_{Iso}	Fraction of single-infected (sick) who are isolated (or home isolated)	48%
$\tilde{f}_{\mathrm{Iso}}$	Fraction of multi-infected (sick) who are isolated (or home isolated)	52.8%
f_{Dead}	Fraction of single-infected (sick) who die from the disease	4%
$\tilde{f}_{\mathrm{Dead}}$	Fraction of multi-infected (sick) who die from the disease	5%

Summary of parameters describing the number of Erlang states, durations, transition rates, morbidity and mortality, and their default parameter choices.
