# Supplementary material for: Is increased mortality by multiple exposures to COVID-19 an overseen factor when aiming for herd immunity?
Source: PLoS One. 2021 Jul 16;16(7):e0253758. doi: 10.1371/journal.pone.0253758 (PMC8284653; doi:10.1371/journal.pone.0253758)
Supplement: S3 Table — (PDF) [file pone.0253758.s006.pdf]

S3 Table. Summary of model parameters describing infections and choices for the simulations.

Name	Description	Value/Eq.
$\lambda(t)$	Total force of infection	$\lambda^{(\mathrm{s})}(t)+\lambda^{(\mathrm{m})}(t)$
$\lambda^{(\mathrm{s})}(t)$	Force of infection leading to single-infection	Eq.(22)
$\lambda^{(\mathrm{m})}(t)$	Force of infection leading to multi-infections	Eq. (23)
$\lambda^{(\mathrm{s})}_{\mathrm{Ext}}$	External force of infection leading to single-infections	50/day
$\lambda^{(\mathrm{m})}_{\mathrm{Ext}}$	External force of infection leading to multi-infections	0
$\tilde{m}$	Fraction of contacts with multi-infected that cause multi infections	0.2
q_E	Prob. that single-infected lead to transient multi-infection in latent states	1
q_P	Prob. that single-infected lead to transient multi-infection in prodromal states	0.75
q_I	Prob. that single-infected lead to transient multi-infection in fully contagious states	0.5
q_L	Prob. that single-infected lead to transient multi-infection in late-infectious states	0.25
$\bar{R}_0$	Annual average basic reproduction number	3.2
a	Amplitude of the seasonal fluctuation of the basic reproduction number	0.35
$t_{R_{0_{\max}}}$	Day when R_0 reaches its maximum	335
c_P	Relative contagiousness in the prodromal period	0.5
c_I	Relative contagiousness in the fully contagious period	1
c_L	Relative contagiousness in the late infectious period	0.5
$\beta_P(t)$	Seasonally varying effective contact rate of prodromal ind.	Eq. (18)
$\beta_I(t)$	Seasonally varying effective contact rate of full contagious ind.	Eq. (19)
$\beta_L(t)$	Seasonally varying effective contact rate of late-infectious ind.	Eq. (20)

Summary of parameters describing infectiousness, contact rates, forces of infection, and their default parameter choices.
