# Supplementary material for: Is increased mortality by multiple exposures to COVID-19 an overseen factor when aiming for herd immunity?
Source: PLoS One. 2021 Jul 16;16(7):e0253758. doi: 10.1371/journal.pone.0253758 (PMC8284653; doi:10.1371/journal.pone.0253758)
Supplement: S4 Table — (PDF) [file pone.0253758.s007.pdf]

S4 Table. Summary of model parameters and choices for the simulations.

Name	Description	Value
Q_{max}	Maximum capacity of the isolation units per 10 000	30
p_{Home}	Prevented fraction of contacts of individuals who are isolated at home	0.75
p_{Dist}	Prevented fraction of contacts because of general social-distancing measures	0.4
t_{Iso_1}	Day when the case-isolation measures start in the population	day 20
t_{Iso_2}	Day when the case-isolation measures end in the population	day 900

Summary of parameters describing interventions and default parameter choices.
