# Supplementary material for: Is increased mortality by multiple exposures to COVID-19 an overseen factor when aiming for herd immunity?
Source: PLoS One. 2021 Jul 16;16(7):e0253758. doi: 10.1371/journal.pone.0253758 (PMC8284653; doi:10.1371/journal.pone.0253758)
Supplement: S5 Table — (PDF) [file pone.0253758.s008.pdf]

S5 Table. Contact reduction parameters (reflecting roughly the policies in the USA).

Initial day	$[t_{\mathrm{Dist}_i}, t_{\mathrm{Dist}_{i+1}}]$	p_{Cont_i}	Description
2020-10-03	$[50, 115]$	55%	Interval of first contact restrictions (e.g. travel bans etc.)
2020-05-14	$[115, 190]$	22%	Interval where restrictions were partly lifted
2020-07-28	$[190, 255]$	55%	Interval of the first "hard lockdown"
2020-10-01	$[255, 290]$	45%	Interval of "soft lockdown"
2020-11-05	$[290, 309]$	65%	Interval of second "hard lockdown"
2020-11-24	$[309, 316]$	55%	Interval of second "relief period"
2020-12-01	$[316, 325]$	60%	Interval of "hard lockdown"
2020-12-10	$[325, 335]$	70%	Interval of stronger "hard lockdown"
2020-12-20	$[335, 354]$	55%	Interval of "soft lockdown"
2021-01-08	$[354, 450]$	65%	Interval of "hard lockdown"
2021-04-14	$[450, 900]$	0%	Interval when general contact reduction ends

Summary of parameters describing interventions and default parameter choices reflecting roughly the policies in the USA) in the first year of the epidemic and hypothetical reductions afterwards.
