# Supplementary material for: Is increased mortality by multiple exposures to COVID-19 an overseen factor when aiming for herd immunity?
Source: PLoS One. 2021 Jul 16;16(7):e0253758. doi: 10.1371/journal.pone.0253758 (PMC8284653; doi:10.1371/journal.pone.0253758)
Supplement: Attachment — Submitted filename: Reply to reviewer.pdf [file pone.0253758.s009.pdf]

Responses to Review Comments for the manuscript with number [PONE-D-20-32872] - [EMID:1556dbe9ef78511d], entitled "Is increased mortality by multiple exposures to COVID-19 an overseen factor when aiming for herd immunity?"

Reviewers and suggested responses

Reviewer #1:

Comment 1: There are many constants relating to the disease spread that are assumed throughout the study and the authors should provide a rationale for most of them or some reference to why these were chosen.

Response: This is an important remark. Overall, our model's aim is to serve as a preparedness tool that helps policy-making in the management of the pandemic, with a focus on the effect of multi-infections. In the revised version all simulations have been updated, as we had the feeling those from the original submission were outdated. The new simulations reflect the situation in the USA as an example. We used parameter choices that accurately captured the dynamics in the first year of the epidemic. While some parameters are known (such as population size, quarantine units, etc.), estimates of other parameters such as the duration of the infectious stages exist as they can be obtained from clinical data. In the model, parameters reflecting contact reduction were estimated from data on contact behavior. We added a statement at the end of the introduction to clarify the fact that contact reduction parameters used to describe future lockdowns are hypothetical as they cannot be predicted. Moreover, we added a statement with references in the result section to further clarify how the parameters were chosen.

However, it is important to notice that some of the parameters cannot be estimated from real-world data, e.g., parameters related to multi-infections. These were chosen hypothetically for a plausible range as the ms's main purpose is to study their effects.

Comment 2: Some of the legends are missing in the figures (such as the dashed lines) and these should be added.

Response: We appreciate the reviewer for raising this concern. All figures have been updated and we added the missing legends. Unfortunately, there is no perfect solution: legends symbolizing different levels are given in different colors in the first panel of the figures and those for the line-types are in the second panel (these have been added). To increase visibility, we decided to include the legend only in one panel. A dashed grey line indicates seasonal fluctuations in R0; grey shading indicates the intensity of general distancing in the respective time intervals - this is only explained in the caption. We hope the figures satisfactory address the concerns raised.

Comment 3: The disease caused by the virus is very age dependent. However, the authors did not give any consideration to different probabilities due to different ages. This should at least be discussed in the discussion section. See for example models Mizrahi, Stern, Open Biology. Balabdaoui, Mohr Scientific Reports.

Response: Age-dependence is indeed important. Particularly, if the model is applied to countries in which age structure cannot be neglected. However, this is a limitation that is now addressed in the discussion. We neglected age structures for the following reasons: (i) We had mainly industrial nations in mind, in which the herd immunity strategy was particularly controversial since these countries have the financial means to implement strong and efficient response strategies to COVID-19. (ii) We fully agree that age is most relevant for the progression of the disease. However, we considered the average proportion of being symptomatic or having severe infections. In

populations in which age structure can be neglected this is sufficiently accurate. In general, age structure can be modelled explicitly in a rather simple way. Important is that the contact behavior between age groups is modelled explicitly (not assumed to be random - such a model would be equivalent to a model without age dependency, but stratified into age groups). (iii) The model itself would be much more complicated as R0 has to be defined by the next-generation matrix (which can be done rather easily here), but the description becomes much more complex. Our aim was to show the potential dangers of herd-immunity strategies. Age-dependency is not necessary to show this effect.

Comment 4: Table 2 is hard to understand what each column represents.

Response: All simulations were updated. Particularly, the new simulations show that the model can be parameterized to reflect real-world situations. Since the original submission, several contact-reducing interventions have been imposed in the USA. We decided to use the policies in the US as an example and chose the parameters accordingly. For the first 450 days, only one scenario is assumed. To investigate possible outcomes in the future, alternative intensities of general distancing after day 450 were simulated. Future contact reductions cannot be anticipated as these are dependent on disease incidence. We, therefore, assumed a number of different contact reductions for the future. Table 2 was removed due to updated simulations as it became unnecessary. Now, the alternative scenarios tested only differ by the strength of general distancing after day t=450, which should be easier to understand; the results are presented in Fig 7. We believe the updated results are easier to understand and show more realistic dynamics.

Comment 5: Ro is relatively high for todays' measures of social distancing and face masks. The authors address this in the discussion but it would be good to perhaps add in the supplementary a scenario where social distancing and masks are kept.

Response: We understand the reviewers' concern and we are aware of the fact that the formal definition of R0 and the quantities reported in the media diverge. We refer to R0 in the strict classical definition, i.e. as the number of secondary infections per primary infection in a completely susceptible population. R0 does not change with the number of infections nor with contact reducing measures. The quantity that changes is the effective reproductive number RE, which is typically referred to as R0 in the media - although incorrect in the strict sense. In our model, the effective reproductive number is much lower than R0 since we assume contact reductions. The effects of wearing facial masks etc. are subsumed into general contact reductions. We updated all simulations. In those that were presented in the supplement, we now use scenarios with different durations in which general distancing (e.g. facial masks) are sustained, particularly some in which general distancing is sustained for prolonged time periods. In the revised version we make the distinction between R0 and RE explicit.

Comment 6: The reduction in the interactions in lock down scenarios is relatively mild (40%-60%). What happens in a more tight lock down?

Response: See also responses to points 4 and 5. We found our original simulations outdated and adjusted them such that they appropriately reflect the situation in the US. In the US contact reductions were relatively mild compared with other countries, e.g., Australia, South Korea, Germany, etc. We believe the updated simulations are inline with the intention of this comment. The reduction of 40-60% seems mild, but actually, it is not. The parameters chosen were derived from estimates on age-dependent contact behavior (at work, in schools, at home, and at other places). A majority of contacts take place at home. Contact reductions higher than 75% imply a strong lockdown that affects even the contact behavior at home. The harder a lockdown, the more the delay of epidemic outbreaks.

Comment 7: How likely is a multi-infection scenario given that most people stay within their area in these times and at the same area usually there is the same strain of the virus? Maybe it would be good to add references about local different strains.

Response: The reviewer's comment is definitely justified. A multi-infection also reflects higher viral loads upon infection due to multiple contacts. There are definitely viral variations as reflected by the novel mutations reported in the media. The variants reported are characterized by different reproductive numbers. This is ignored in the model. The rationale in the model is that if the virus is at low prevalence, infections are always introduced from "outside the area", otherwise there would be no virus. If the prevalence is high, the number of viral variants circulating increases substantially. In situations of intermediate prevalence, multi-infections are negligible anyway. Therefore, the model will accurately reflect what we have in mind. We added at the end of the discussion a statement on the UK variant that illustrates our argument. As suggested, we added more references about the diversity of viral variants in the introduction.
