# Supplementary material for: Is increased mortality by multiple exposures to COVID-19 an overseen factor when aiming for herd immunity?
Source: PLoS One. 2021 Jul 16;16(7):e0253758. doi: 10.1371/journal.pone.0253758 (PMC8284653; doi:10.1371/journal.pone.0253758)
Supplement: Attachment — Submitted filename: Reply to reviewer.pdf [file pone.0253758.s010.pdf]

**Responses to Review Comments for the manuscript with number [PONE-D-20-32872R1] - [EMID:97047208cf9005eb], entitled "Is increased mortality by multiple exposures to COVID-19 an overseen factor when aiming for herd immunity?"**

**Editor/Reviewers comments and suggested responses**

**Editor:**

I would like you to verify the accuracy and relevance of all your references.

**Response:** The bibliography was indeed in extremely bad shape and inadequate for publication. We verified the accuracy of all references, updated, changed, and added references as appropriate. We went carefully through all references and fixed them according to the submission guidelines.

Some references like 19 and 20 seem to be irrelevant to the points stated in the text.

**Response:** We agree that reference [19] was irrelevant. The sentence was slightly adapted and the references were replaced by a more recent and relevant one. We decided to keep reference [20], but added two additional references and slightly modified the text.

Ref 24 is published in PloS One and does not describe ADE but uses it in the model.

**Response:** We appreciate the observation, indeed the reference was inappropriate at one place in the discussion, where it was removed. The reference per se was kept, as it is referred to at other places, in the appropriate context.

Reference is required for the following statements: "Increased viral load might be caused by multiple infectious exposures during the course of a COVID-19 episode", and "Such infections can lead to a higher viral load upon infection or super-infections with different viral variants during the course of the disease."

**Response:** We agree, we provided references for these statements (which now exist), and the text was slightly modified.

Some references like 16 and 17 are outdated. More relevant papers have been recently published about genetic diversity.

**Response:** We agree. We deleted the old references and replaced them by more recent ones.

Some references like 31 are incomplete

**Response:** All references were checked for their completeness. The shape of the bibliography was indeed bad. It has now been corrected and double checked.

**Reviewer #1:**

**Comment 1:** All comments have been addressed.

**Response:** We are happy!
